# Advanced metastatic pancreatic neuroendocrine tumor treated successfully with peptide receptor radionuclide therapy: a case report

**DOI:** 10.37349/etat.2022.00089

**Published:** 2022-06-29

**Authors:** Amit Kumar, Shweta Tanwar, Sudhish Gupta, Rajesh Chetiwal, Rohit Kumar

**Affiliations:** 1 Department of Medicine, ESIC Postgraduate Institute of Medical Sciences and Research, Basaidarapur, New Delhi 110015, India; 2 Scientist C, Indian Council of Medical Research, New Delhi 110029, India; 3 Department of Pharmaceutical Sciences, Maharshi Dayanand University, Rohtak, Haryana 124001, India; Humanitas University, Humanitas Research Hospital, Italy

**Keywords:** Neoplasm metastasis, neuroendocrine tumors, pancreas, peptide receptor radionuclide therapy

## Abstract

Neuroendocrine tumor (NET) is a rare tumor that has been observed in different sites such as lungs and throughout the gastrointestinal tract. Clinical features are usually non-specific and vary considerably depending upon the location of the tumor. Symptoms are similar to those of common conditions such as peptic ulcer disease, gastritis, irritable bowel syndrome, asthma, etc. Thus, an initial diagnosis of a NET usually occurs at an advanced stage. This report describes a case of pancreatic NET (PNET, grade 2) with liver metastasis in a 37-year-old male which was found to be inoperable due to extensive direct involvement of the proximal jejunal branches and superior mesenteric vein. Peptide receptor radionuclide therapy (PRRT) with lutetium-177 dotatate (^177^Lu-DOTATATE) was administered due to the inoperability of primary PNET. Complete resolution of symptoms occurred with three cycles of PRRT.

## Introduction

Neuroendocrine tumors (NETs) are rare type of neoplasms derived from specialized cells known as enterochromaffin cells. They are most commonly found in the gastro-intestinal tract and lungs and can be benign or malignant [1]. The most common sites within the gastro-intestinal tract are small intestine, rectum, and colon followed by pancreas, stomach, and appendix [2]. Metastasis from NETs frequently involves liver, lungs, bone, and adrenals [3]. Surgery remains the mainstay of treatment. The present report highlights a case of pancreatic NET (PNET) with metastasis to the liver and was found to be inoperable due to direct extensive invasion of proximal jejunal branches and superior mesenteric vein. As PNET is a very rare tumor, this report presents the diagnostic and therapeutic challenges in the management of such a tumor.

## Case report

A 37-year-old man [body mass index (BMI) 22.7 kg/m^2^] presented with chronic diarrhea for one year. He complained of watery, non-bloody diarrhea which was sometimes associated with urgency. He reported 5–8 episodes of moderate-sized bowel movements per day without a nocturnal component. He denied blood in stool, abdominal pain, fever, or fecal incontinence. However, he had anorexia and weight loss for the past year. There was no complaint of nausea, vomiting, jaundice, dyspnea, or wheezing. He had taken antibiotics several times but didn’t have any improvement. His symptoms were not associated with any dietary triggers; however, he had been advised to eliminate milk products, caffeine, and gluten from his diet, which he did but with no improvement. Physical examination revealed normal thyroid and no hepatosplenomegaly. No abnormality was detected on systemic clinical examination.

**Figure 1.**
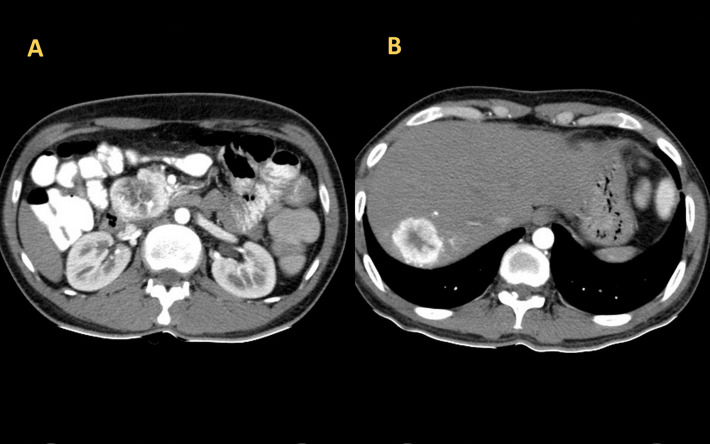
Axial section image of CT scan showing heterogeneously enhancing mass lesion in the head of pancreas (A) and right lobe of liver (B)

His initial laboratory studies demonstrated a hemoglobin of 14.1 g/dL, a total leucocyte count of 4,200/cumm, and a platelet count of 1.72 × 10^5^/cumm. Liver and renal function tests were unremarkable. Thyroid function test and blood glucose were within normal limits. Serum immunoglobulin A (IgA) anti-tissue transglutaminase and IgA anti endomysial antibodies were negative. Abdominal ultrasonography revealed a hypoechoic lesion in the liver with retroperitoneal lymphadenopathy. Triple phase contrast-enhanced computed tomography (CT) of abdomen revealed a well-defined mass lesion of size 40 mm × 47 mm × 48 mm in close relation to the head of pancreas and another well-defined lesion of size 48 mm × 33 mm in segment VII of the right lobe of liver with both lesions showing arterial phase enhancement (Figure 1). Serum alphafeto protein (AFP) was 4.59 IU/mL, carbohydrate antigen (CA) 19-9 was 176.42 IU/mL, and carcinoembryonic antigen (CEA) was 1.26 ng/mL. Serology for hepatitis B and C viruses, herpes simplex virus, cytomegalovirus, and epstein-barr virus were negative. Serum chromogranin-A and neuron-specific enolase were elevated with values of 182.10 ng/mL and 27.71 ng/mL respectively. Serum gastrin, vasoactive intestinal peptide, and insulin levels (fasting and postprandial) were within normal limits. The 24 h urinary level of 5-hydroxyindolacetic acid (5-HIAA) was found to be increased.

**Figure 2.**
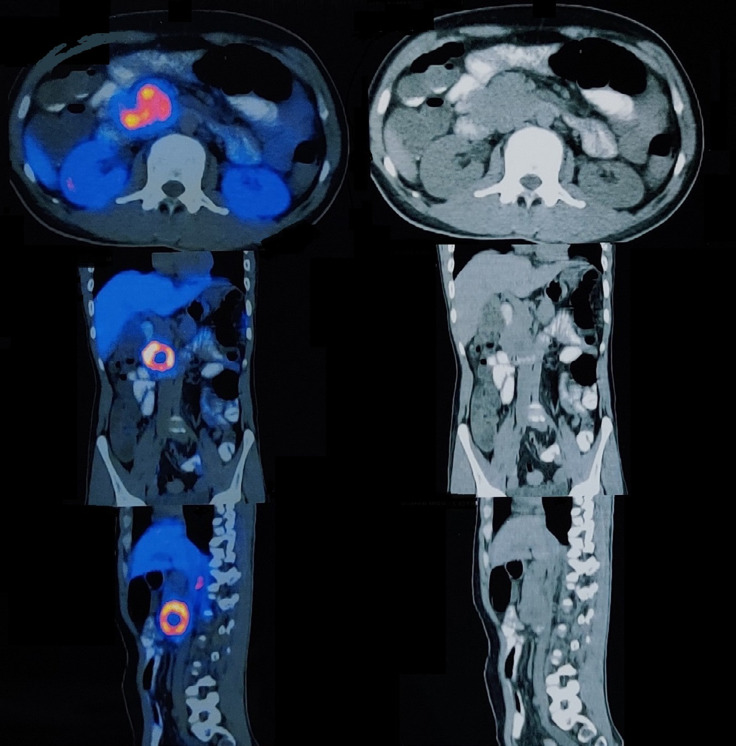
Axial, coronal, and sagittal section images of ^68^Ga DOTANOC PET CT scan showing enhancing lesion in the head of pancreas

**Figure 3.**
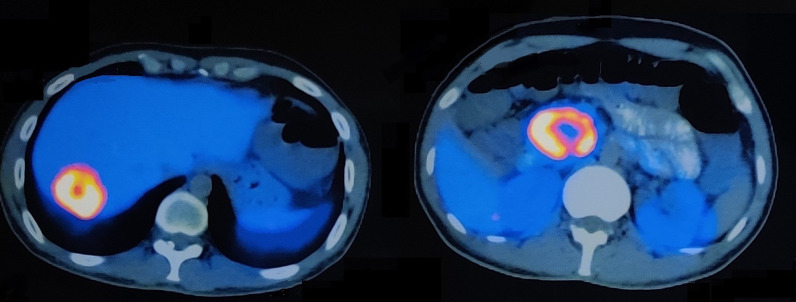
Axial section images of ^68^Ga DOTA-NOC PET CT scan showing enhancing lesions in the segment VII of liver (SUV max-14.7) and head of pancreas (SUV max-38.2)

The ^68^Ga DOTA-NOC PET CT scan [somatostatin receptor (SSTR) PET CT] showed active SSTR expressing lobulated, centrally necrotic mass lesion with intense DOTA-NOC avidity [standardized uptake value (SUV) max-38.2] involving the head of pancreas measuring approximately 43 mm × 50 mm × 48 mm, closely abutting the duodenum and inferior vena cava (Figure 2). Another active SSTR expressing tracer avid lesion (SUV max-14.7) was noted in segment VII of liver measuring 40 mm × 38 mm, suggestive of metastatic disease (Figure 3). Biopsy done from the lesion in liver depicted metastatic well-differentiated NET, favoring PNET-grade 2. On immunohistochemistry, Ki-67 proliferation index was around 6% and tumor cells showed diffuse cytokeratin, synaptophysin, and chromogranin expression. Based on the clinical, laboratory, histopathologic, and radiological findings, a diagnosis of PNET with metastasis to the liver was made. The patient was planned for surgery.

On diagnostic laparoscopy, there was a single metastatic lesion in segment VII of the liver with the rest of the peritoneal cavity free of metastatic disease. There was a direct invasion of the proximal jejunal branches and superior mesenteric vein by the tumor. The procedure was converted to open laparotomy. Common hepatic lymph node dissection, hepatic metastasectomy, cholecystectomy, and kocherization of the duodenum were done. Jejunal mobilization was tried, but in view of direct extensive invasion by the tumor of proximal jejunal branches, the procedure couldn’t be proceeded and abandoned. Due to the inoperability of the primary tumor, the patient was treated with peptide receptor radionuclide therapy (PRRT) with lutetium-177 dotatate (^177^Lu-DOTATATE). The patient received three cycles of PRRT with ^177^Lu-DOTATATE. At 9 months follow-up, the patient reported complete resolution of symptoms with improved quality of life. Biochemical markers status also showed stable disease with serum chromogranin-A and neuron-specific enolase reported values of 97 ng/mL and 15.6 ng/mL respectively. The post-treatment ^68^Ga DOTA-NOC PET CT scan could not be performed as the patient had already incurred a huge financial burden and could not afford the costly imaging.

## Discussion

PNETs comprise approximately 7% of all NETs and only 1–2% of all pancreatic tumors [2, 4]. There has been a significant rise in the incidental diagnosis of PNETs over the last decade owing to the extensive use of advanced diagnostic imaging modalities. While a majority of PNETs are sporadic, around 10–30% develop in association with some genetic syndrome, the most common of which is multiple endocrine neoplasia (MEN) type 1 followed by MEN type 4, von Hippel-Lindau disease, neurofibromatosis 1 (von Recklinghausen’s syndrome), and tuberous sclerosis [5]. PNETs are classified as non-functional and functional. A majority (up to 90%) of PNETs are non-functional and do not produce excess hormones. Therefore, they often grow to a large size before causing non-specific symptoms such as abdominal pain, loss of appetite, and weight loss. On the other hand, functional PNETs produce excess hormones causing specific clinical features and include insulinomas, glucagonomas, gastrinomas, somatostatinomas, VIPomas, and adrenocorticotropic hormone secreting tumors. Most of the functioning NETs (up to 70%) are insulinomas whereas others are much less common. The most commonly used neuroendocrine markers for PNETs include chromogranin A, neuron-specific enolase, pancreastatin, and pancreatic polypeptide. Combined testing for both chromogranin A and neuron-specific enolase considerably increases the diagnostic power. For diagnosis of a functional tumor, an appropriate hormone evaluation should also be done. Amongst radiological imaging, SSTR-based imaging (SRI) has proved to be the most important in the diagnosis and staging of PNETs. Mitotic count and Ki-67 expression are considered to be significant on histopathological examination. In 2017, World Health Organization (WHO) revised the grading classification for PNETs to improve the prediction of clinical outcomes and to determine better therapeutic strategies and patient care. The revised system classified PNETs into well-differentiated PNETs consisting of G1 PNETs [< 2 mitoses per 10 high power fields (HPFs) and a Ki-67 proliferation index < 3%], G2 PNETs (between 2 and 20 mitoses per 10 HPFs or a Ki-67 proliferation index ranging between 3% and 20%) and G3 PNETs (> 20 mitoses per 10 HPFs or a Ki-67 proliferation index > 20% without poorly-differentiated pathological features), and poorly-differentiated PNETs which constitute G3 PNETs having > 20 mitoses per 10 HPFs or a Ki-67 proliferation index > 20% with poorly-differentiated small cell or large cell features [6].

From a treatment perspective, surgery is the therapy of choice for functional tumors irrespective of the tumor size. The surgical therapy, whenever feasible offers the best option to cure the syndromes and improve the disease outcome [7]. The National Comprehensive Cancer Network (NCCN) guidelines recommend tumor enucleation with peri duodenal lymph node dissection for peripheral tumors in the head of pancreas and pancreato-duodenectomy for deeper, invasive tumors. Distal pancreatectomy with the removal of peritumoral lymph nodes is recommended for distal pancreatic tumors [8]. While for larger non-functional tumors, surgery remains the treatment of choice, the guidelines are controversial to recommend surgery as the primary treatment for a PNET ≤ 2 cm. There has been a growing inclination toward the role of observation and active surveillance in the case of small asymptomatic non-functional PNETs (≤ 2 cm) considering the mortality, morbidity, and long-term complications of pancreatic surgery [9]. For advanced disease, the NCCN guidelines recommend complete resection of the primary tumor and metastases, wherever feasible and non-curative debulking may be performed in selected advanced cases for symptom control and to prolong survival [8]. In the present case, surgical resection of the primary tumor could not be performed due to extensive direct invasion of proximal jejunal branches. Treatment options for such inoperable tumors include somatostatin analogues and PRRT. Because of the rarity of PNETs, randomized controlled studies have not been done and current treatment recommendations are based primarily on case series and individual treatment approaches. The recommendation of PRRT therapy for gastroenteropancreatic (GEP) NETs was primarily based on the NETTER-1 trial. However, the NETTER-1 trial enrolled patients with small bowel NETs only and did not include PNETs [10]. Symptomatic improvement with tumor regression occurs with PRRT such as ^177^Lu-labelled somatostatin analogues. Studies with ^177^Lu-DOTATATE indicate that the therapy is safe, effective, has no serious side effects and the median progression-free survival is longer than 40 months [11]. It also improves the quality of life significantly as reported in the present case. The repeated cycles of PRRT are safe and enable stabilization of the disease without causing significant toxicity. PRRT therapy might become the first-line therapy in patients with disseminated or inoperable GEP NETs [12]. The assessment of response to PRRT is challenging as there has been no consensus on the method and timing of evaluation. In recent years, the evaluation of therapy outcome has focussed more on the quality of life as several studies have suggested it to be an important prognostic parameter for PRRT response with improved quality of life significantly associated with overall survival.

This case report highlights the diagnostic and therapeutic challenges in PNETs. Due to the wide heterogeneity of the presentation of the disease, a high index of clinical suspicion is important for early diagnosis. Although surgery is the mainstay of treatment but is not feasible in advanced cases. The treatment of advanced NETs should be individualized based on the prognostic and predictive factors and the availability of novel treatment modalities. PRRT is an emerging treatment option in advanced and inoperable NETs improving patient outcomes and overall quality of life.

## Abbreviations

^177^Lu-DOTATATE: lutetium-177 dotatate
CT: computed tomography
HPFs: high power fields
NET: neuroendocrine tumor
PNET: pancreatic neuroendocrine tumor
PRRT: peptide receptor radionuclide therapy
SSTR: somatostatin receptor
SUV: standardized uptake value

## Declarations

### Copyright

© The Author(s) 2022.
